# Complete chloroplast genome sequence of *Barleria prionitis*, comparative chloroplast genomics and phylogenetic relationships among Acanthoideae

**DOI:** 10.1186/s12864-020-06798-2

**Published:** 2020-06-06

**Authors:** Dhafer A. Alzahrani, Samaila S. Yaradua, Enas J. Albokhari, Abidina Abba

**Affiliations:** 1 Department of Biological Sciences, King Abdulaziz University, Jeddah, Saudi Arabia; 2 Department of Biology, Umaru Musa Yaradua University, Centre for Biodiversity and Conservation, Katsina, Nigeria; 3 Department of Biological Sciences, Umm Al-Qura University, Makkah, Saudi Arabia

**Keywords:** Acanthoideae, Chloroplast genome, *Barleria prionitis*, Phylogenomics

## Abstract

**Background:**

The plastome of medicinal and endangered species in Kingdom of Saudi Arabia, *Barleria prionitis* was sequenced. The plastome was compared with that of seven Acanthoideae species in order to describe the plastome, spot the microsatellite, assess the dissimilarities within the sampled plastomes and to infer their phylogenetic relationships.

**Results:**

The plastome of *B. prionitis* was 152,217 bp in length with Guanine-Cytosine and Adenine-Thymine content of 38.3 and 61.7% respectively. It is circular and quadripartite in structure and constitute of a large single copy (LSC, 83, 772 bp), small single copy (SSC, 17, 803 bp) and a pair of inverted repeat (IRa and IRb 25, 321 bp each). 131 genes were identified in the plastome out of which 113 are unique and 18 were repeated in IR region. The genome consists of 4 rRNA, 30 tRNA and 80 protein-coding genes. The analysis of long repeat showed all types of repeats were present in the plastome and palindromic has the highest frequency. A total number of 98 SSR were also identified of which mostly were mononucleotide Adenine-Thymine and are located at the non coding regions. Comparative genomic analysis among the plastomes revealed that the pair of the inverted repeat is more conserved than the single copy region. In addition high variation is observed in the intergenic spacer region than the coding region. The genes, *ycf1*and *ndhF* and are located at the border junction of the small single copy region and IRb region of all the plastome. The analysis of sequence divergence in the protein coding genes indicates that the following genes undergo positive selection (*atpF, petD, psbZ, rpl20, petB, rpl16*, *rps16, rpoC, rps7, rpl32* and *ycf3*). Phylogenetic analysis indicated sister relationship between Ruellieae and Justcieae. In addition, *Barleria*, *Justicia* and *Ruellia* are paraphyletic, suggesting that Justiceae, Ruellieae, Andrographideae and Barlerieae should be treated as tribes.

**Conclusions:**

This study sequenced and assembled the first plastome of the taxon *Barleria* and reported the basics resources for evolutionary studies of *B. prionitis* and tools for phylogenetic relationship studies within the core Acanthaceae.

## Background

The Acanthaceae Juss. Ex Bercht.& J. Presl is among the largest family in the order Lamiales with ca. 3800 recognized species accommodated in ca. 200 genera [1], the members of the family are mainly diversified in the sub tropics and tropics, with few species in the temperate zones [2]. The family is close to Bignoniaceae family in the Lamiales order [3]. The main centres of distribution of the species in the family are Africa, Central America and Asian continent particularly Malaysia, Indonesia and Brazil [4]. They are characterized by having decussate phyllotaxis, while some species have congest whorled phyllotaxis, the leaves are usually simple with toothed margin, opposite, existipulate and contained calcium oxalate crystals or hypodermal calcium carbonate cystolith [5, 6].

In an effort to resolve taxonomic issues of the family and its species, researchers for the past decades works extensively in delimiting the family [7–10], identifying major clades in the family [11–14]. Scotland and his colleagues carried out infrafamilial studies using floral parts [15–17], their findings gives more insight on the infra familial classification of the family and gives morphological synapomorphies of the major lineages. Recently, phylogenetic approach was used to reveal the relationships between the lineages [18–20]. Despite these researches, the classifications of the species within the Acanthoideae are still not clear.

The chloroplast organelle is one the most distinguishing featured that differentiates plant cell and other type of cells; therefore it is the most noticeable feature in plants. The organelle which is semi-autonomous is believed to have evolved decade of millions years ago from cynobacterium [21, 22]. The plastome of flowering plant is conserved than the other genomes (i.e mitochondrial and nuclear genomes), in addition the genome is small compared with the others and it is used frequently in phylogeny studies due to its low rate of nucleotide substitution [23]. The chloroplast genome is typically quadripartite in structure, containing large single copy (LSC) and small single copy (SSC) separated by pair of inverted repeat (IR) [24]. The genome organization, its content and gene structure are highly conserved [25]. Due to its conserved nature, the cp genome contents are widely used by researchers as a tool to investigate phylogenetic relationship and in genomic studies [26]. Single nucleotide polymorphisms as well as insertion/deletions which are among the evolutionary hotspot of the organelle are believed to be use as a tool to solve taxonomic issues among taxa that their phylogenetic relationships are unresolved. Phylogenetic relationship generated from single or combination of few genes are being replaced by the ones constructed from the whole genome as a result of new DNA sequencing methods such as next generation sequencing (NGS). The introduction of next generation sequencing has increased the availability of data for solving phylogenetic relationship issues. However, in spite of its importance, the approach is not fully and well utilize by researchers in plant systematic studies [27–29]. One of the most important benefits of next generation sequencing technique is that it generate very high amount of sequences compared with sanger sequencing technique. Additionally, the platform used in next generation sequencing like Illumina is very cheap process [30]. This approach has been used to generate huge number of data for inferring phylogenetic relationship in different taxonomic levels inference [31–34].

With the advent of next generation sequencing, importance of plastome sequence in resolving phylogenetic relationships and the great number of genera in Acanthaceae, only plastome of few genera have been sequenced and no phylogenomic studies have been conducted for the family.

In this research, we sequenced and characterized the plastome of *Barleria prionitis* and compared the genome with cp genomes from Acanthoideae species. We used data from the whole chloroplast genome of 8 genera belonging to the Acanthoideae to reveal their tribal positions. This is as a result of incongruent of previous studies in placing the genera in their respective tribes [35]. placed Barlerieae and Andrographideae as sub tribes under the tribe Justicieae, this classification has been reported by other student of Acanthaceae [27]. classify the sub family Acanthoideae into two tribes, placing Ruelliinae, Justiciinae, Andrograpiinae and Barleriinae under the tribe Ruellieae. Findings of recent studies by McDade and her colleagues using molecular data contradict with previous classifications. Therefore, there is need to use complete chloroplast genome to address the correct placement of the genera into their respective tribes. The result of this study will be useful for developing makers, provide resources for evolutionary studies and authentication of *B. prionitis* and the inference of phylogenetic relationships within Acanthoideae.

## Results

### Characteristics of *B. prionitis* chloroplast genome

**Fig. 1:**
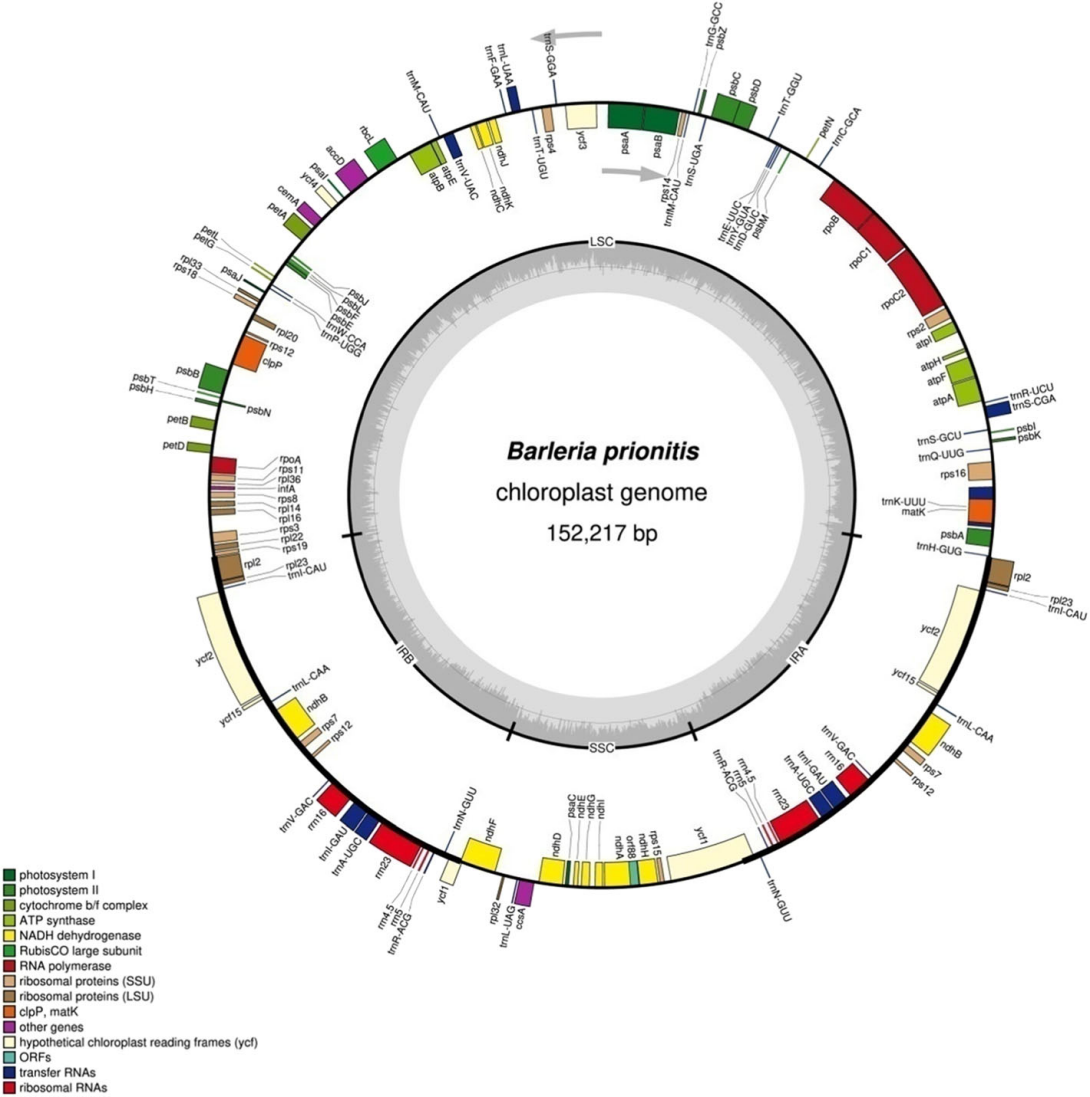
Gene map of the *B. prionitis* chloroplast genome. Genes outside the circles are transcribed in counter clockwise direction and those inside in clockwise direction. Known functional genes are indicated in the colored bar. The GC and AT content are denotes by the dark grey and light grey colour in the inner circle respectively. LSC indicates large single copy; SSC, indicates small single copy and IR, indicates inverted repeat

The complete plastome sequence of *B. prionitis* was reported to be 152,217 bp in size and has a structural organization of quadripartite containing a large single copy (LSC, 83, 772 bp), a pair of inverted repeat (IRa and IRb 25, 321 bp each) and small single copy (SSC, 17, 803 bp) (Fig. 1 and Table 1). Composition of Adenine-Thymine and Guanine-Cytosine content in *B. prionitis* was 61.7 and 38.3%, respective whereas the IRA, IRB, SSC and LSC regions have, 67.4 and 32.6%, 56.5 and 43.5%, 56.4 and 43.6%, and 63.6% and 36.4, respectively (Table 1). The inverted repeat region have higher GC content of 49% compared with the SSC and LSC regions with 32.6 and 36.4% respectively (Table 1). In terms of the size of the coding and non coding region, the protein coding regions is 79, 950 pb in length whereas the non coding which includes the intergenic spacer and introns have 72, 267 bp.

**Table 1:** Nucleotide composition in the complete plastome sequence of *B. prionitis*

Region		T(U) (%)	C (%)	A (%)	G (%)	Length (bp)
cp Genome		31.2	19.5	30.5	18.8	152,217
LSC		32.4	18.7	31.2	17.7	83,772
SSC		33.6	17.1	33.8	15.5	17,803
IRA		28.2	22.5	28.2	21.0	25,321
IRB		28.2	21.0	28.3	22.5	25,321
	1st Position	30	20.4	30.4	19.0	50,739
	2nd Position	32	18.7	31.3	18.0	50,739
	3rd Position	31	19.5	29.8	19.4	50,739

The complete chloroplast genome of *B. prionitis* contained 113 different genes out of which 18 are duplicated in the IRA and IRB region, totaling 131 genes. The number of rRNA genes, tRNA genes and protein-coding genes in the genome are 4, 30 and 80, respectively (Fig. 1 and Table 2). Four rRNA, seven protein coding and tRNA genes are located in the pair of the inverted repeat region of the plastome whereas the large single copy region harbored 62 protein-coding sequence and 22 tRNA genes, the remaining one tRNA and 12 protein coding genes are located in the single copy region. Among the genes coding for protein, many of them started with the codon ATG while few starts with other codon such as ACG and GTG, this is also reported in other chloroplast genome of angiosperms.

**Table 2:** Genes present in the chloroplast genome of *B. prionitis*

Category	Group of genes	Name of genes
RNA genes	ribosomal RNA genes (rRNA)	rrn5, rrn4.5, rrn16, rrn23
	Transfer RNA genes (tRNA)	*trnH-GUG, trnK-UUU*^*a*^*, trnQ-UUG, trnS-GCU, trnS-CGA*^*a*^*, trnR-UCU,trnC-GCA, trnD-GUC, trnY-GUA, trnE-UUC, trnT-GGU, trnS-UGA, trnfM-CAU, trnG-GCC, trnS-GGA, trnL-UAA*^*a*^*, trnT-UGU, trnF-GAA, trnV-UAC*^*a*^*, trnM-CAU, trnW-CCA, trnP-UGG, trnI-CAU*^*c*^*, trnL-CAA*^*c*^*, trnV-GAC*^*c*^*, trnI-GAU*^*a,c*^*, trnA-UGC*^*a,c*^*, trnR-ACG*^*c*^*, trnN-GUU*^*c*^*, trnL-UAG,*
Ribosomal proteins	Small subunit of ribosome	*rps2, rps3, rps4, rps7*^*c*^*, rps8, rps11, rps12*^*c*^*, rps14, rps15, rps,16*^*a*^*, rps18, rps19*
Transcription	Large subunit of ribosome	*rpl2*^*a,c*^*, rpl14, rpl16, rpl20, rpl22, rpl23*^*a*^*, rpl32, rpl33, rpl36*
	DNA dependent RNA polymerase	*rpoA, rpoB, rpoC1*^*a*^*, rpoC2*
Protein genes	Photosystem I	*psaA, psaB, psaC, psaI,psaJ,ycf3*^*b*^
	Photosystem II	*psbA, psbB, psbC, psbD, psbE, psbF, psbH, psbI, psbJ, psbK, psbL, psbM, psbN, psbT, psbZ*
	Subunit of cytochrome	*petA, petB, petD, petG, petL, petN*
	Subunit of synthase	*atpA, atpB, atpE, atpF*^*a*^*, atpH, atpI*
	Large subunit of rubisco	*rbcL*
	NADH dehydrogenase	*ndhA*^*a*^*, ndhB*^*a,c*^*, ndhC, ndhD, ndhE, ndhF, ndhG, ndhH, ndhI, ndhJ, ndhK*
	ATP dependent protease subunit P	*clpP*^b^
	Chloroplast envelope membrabe protein	*cemA*
Other genes	Maturase	*matK*
	Subunit acetyl-coA carboxylase	*accD*
	C-type cytochrome systhesis	*ccsA*
	Hypothetical proteins	*ycf2*^*c*^*,ycf4, ycf1*^*c*^
	Component of TIC complex	*ycf*^*c*^

^a^ Gene with one intron, ^b^ Gene with two intron and ^*c*^ Gene with copies

The chloroplast genome of *B. prionitis* is found to have intron in some of the genes, like in other species in the Lamiales order [36, 37]. Out of the 113 different genes, 14 of them contain intron (Table 3), six tRNAs and eight protein-coding genes. Four of the genes with intron viz.: *ndhB*, *trnA-UGC, trnI-GAU* and *rpl2* are situated in the inverted repeat region and the other 12 in the large single copy region. *clpP* and *ycf3* are the only genes with two intron, while the other 12 genes have one intron, this is consistent with that of *S. cusia* [36]. *trnK-UUU* is the gene with longest intron with 2460 bp because of the situation of *matK* in the gene.

**Table 3:** Genes with intron in the *B. prionitis* chloroplast genome and length of exons and introns

Gene	Location	Exon I (bp)	Intron I (bp)	Exon II (bp)	Intron II (bp)	Exon III (bp)
*rps16*	LSC	37	865	228		
*atp F*	LSC	143	664	470		
*rpoC1*	LSC	431	786	1619		
*ycf3*	LSC	128	697	227	750	152
*clpP*	LSC	68	747	290	640	227
*rpl2*	IR	392	676	434		
*ndhB*	IR	776	680	755		
*ndhA*	SSC	551	1082	539		
*trnK-UU*	LSC	36	2460	37		
*trnS-CGA*	LSC	31	667	59		
*trnL-UAA*	LSC	36	487	49		
*trnV-UAC*	LSC	37	595	36		
*trnI-GAU*	IR	41	938	34		
*trnA-UGC*	IR	37	806	34		

**Fig. 2:**
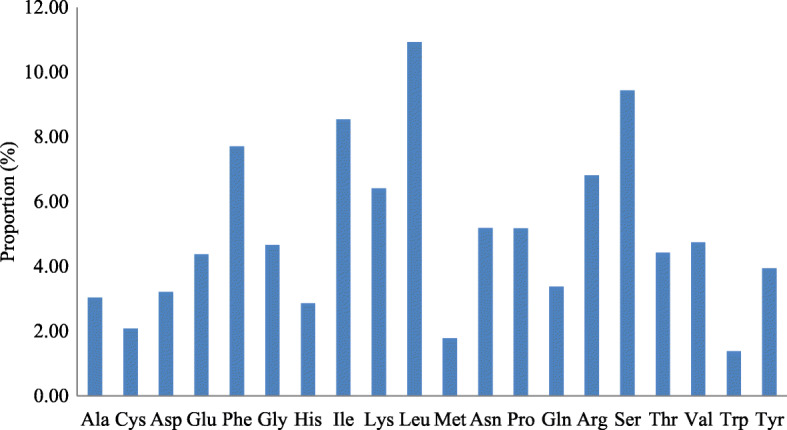
Amino acids frequencies in *B. prionitis* chloroplast genome protein coding sequences

The frequency of the codon usage present in the plastome of *B. prionitis* was computed using the nucleotide sequence of protein-coding genes and tRNA genes 100,319 bp, the result is presented in Table 4, the results showed the genes in the plastome are encoded by 33, 436 codons. The codons that codes for the amino acids Leucine appears more frequently in the genome 3286 (9.83%) (Fig. 2), comparable to that of *Ailanthus altisssima* and the ones coding for Trp have the lowest 622 (1.86%) in the plastid sequence. Guanine-Cytosine ending are more common than the Adenine-Thymine ending, this is incongruent with other cp genome sequence [38–40]. The result of the analysis show that there is low codon usage bias in the plastome sequence of *B.prionitis* (Table 4). 29 codons have RSCU values greater than 1 and all of them are characterized with Adenine-Thymine ending while for 30 codons, were less than 1 and are all of Guanine-Cytosine ending. The amino acids Tryptophan and Methionine have RSCU value of 1 hence they don’t have codon bias.

**Table 4:** Codon – anticodon recognition patterns and codon usage of the *J. flava* chloroplast genome

Codon	Amino Acid	Count	RSCU	tRNA	Codon	Amino Acid	Count	RSCU	tRNA
UUU	Phe	1278	1.18	*trnF-GAA*	UAU	Tyr	964	1.43	*trnY-GUA*
UUC	Phe	882	0.82		UAC	Tyr	384	0.57	
UUA	Leu	704	1.29	*trnL-UAA*	UAA	Stop	556	1.02	
UUG	Leu	717	1.31	*trnL-CAA*	UAG	Stop	484	0.89	
CUU	Leu	660	1.21	*trnL-UAG*	CAU	His	492	1.29	*trnH-GUG*
CUC	Leu	423	0.77		CAC	His	268	0.71	
CUA	Leu	477	0.87		CAA	Gln	685	1.38	*trnQ-UUG*
CUG	Leu	302	0.55		CAG	Gln	309	0.62	
AUU	Ile	1149	1.26	*trnI-GAU*	AAU	Asn	1046	1.39	*trnG-GUU*
AUC	Ile	788	0.86		AAC	Asn	463	0.61	
AUA	Ile	801	0.88	*trnI-CAU*	AAA	Lys	1253	1.29	*trnK-UUU*
AUG	Met	706	1	*trnM-CAU*	AAG	Lys	686	0.71	
GUU	Val	606	1.5	*trnV-GAC*	GAU	Asp	721	1.45	*trnD-GUC*
GUC	Val	258	0.64		GAC	Asp	273	0.55	
GUG	Val	292	0.72		GAA	Glu	943	1.38	*trnE-UUC*
GUA	Val	462	1.14	*trnV-UAC*	GAG	Glu	420	0.62	
UCU	Ser	704	1.45	*trnS-GGA*	UGU	Cys	443	1.19	*trnC-GCA*
UCC	Ser	447	0.92		UGC	Cys	301	0.81	
UCG	Ser	389	0.8		UGA	Stop	595	1.09	
UCA	Ser	614	1.26	*trnS-UGA*	UGG	Trp	622	1	*trnW-CCA*
CCU	Pro	416	1.24	*trnP-UGG*	CGU	Arg	266	0.69	*trnR-ACG*
CCC	Pro	283	0.85		CGC	Arg	148	0.39	*trnR-UCU*
CCA	Pro	393	1.17		CGA	Arg	436	1.14	
CCG	Pro	247	0.74		CGG	Arg	294	0.77	
ACU	Thr	428	1.18		AGA	Arg	761	1.98	
ACC	Thr	311	0.86		AGG	Arg	399	1.04	
ACG	Thr	242	0.67	*trnT-GGU*	AGU	Ser	457	0.94	*trnS-GCU*
ACA	Thr	471	1.3	*trnT-UGU*	AGC	Ser	302	0.62	
GCU	Ala	349	1.34	*trnA-UGC*	GGU	Gly	520	1.05	*trnG-GCC*
GCC	Ala	206	0.79		GGC	Gly	290	0.59	
GCA	Ala	301	1.16		GGA	Gly	670	1.36	
GCG	Ala	186	0.71		GGG	Gly	493	1	*trnG-UCC*

The prediction of RNA editing sites present in the plastome sequence of *B*. *priniotis* was done by means of PREP suite. The first codon of the first nucleotide was used in all the analysis. The results as shown in (Table 5) showed that most of the conversions in the codon positions are from Serine to Leucine. Generally, the editing sites observed in the plastome were 61 which are distributed between the 19 protein-coding genes. *psaB* is found to have the highest number of editing site (13 sites) followed by *ndhB* (9 sites), *rpoB* (6 site) and *rpl20, accD*, *rps, atpI*, *rpl2*, *rpoA* have the lowest number of editing site with 1 editing site each. Nine (9) RNA editing site in *ndhB* has been confirmed in the plastome of other species [41–43]. Conversions of proline to serine were observed, which involves the changing of the amino acids in the RNA editing site from apolar to polar group. Genes such as *petD*, *ndhC*, *atpB*, *clpP*, *ndhE*, *petL, ndhG*, *petG* and *ccsA* among others do not possess RNA editing site in their first codon of the nucleotide.

**Table 5:** Predicted RNA editing site in the *B. prionitis* chloroplast genome

**Gene**	**Nucleotide Position**	**Amino Acid Position**	**Codon Conversion**	**Amino Acid Conversion**	**Score**
*accD*	722	241	TCG = > TTG	S = > L	0.8
*atpF*	791	264	CCC = > CTC	P = > L	1
	914	305	TCA = > TTA	S = > L	1
*atpI*	620	207	TCA = > TTA	S = > L	1
*matK*	469	157	CAC = > TAC	H = > Y	1
	661	221	CAT = > TAT	H = > Y	1
	1264	422	CAT = > TAT	H = > Y	1
*ndhA*	341	114	TCA = > TTA	S = > L	1
	566	189	TCA = > TTA	S = > L	1
**Gene**	**Nucleotide Position**	**Amino Acid Position**	**Codon Conversion**	**Amino Acid Conversion**	**Score**
	1073	358	TCC = > TTC	S = > F	1
*ndhB*	149	50	TCA = > TTA	S = > L	1
	467	156	CCA = > CTA	P = > L	1
	586	196	CAT = > TAT	H = > Y	1
	737	246	CCA = > CTA	P = > L	1
	746	249	TCT = > TTT	F = > F	1
	830	277	TCA = > TTA	S = > L	1
	836	279	TCA = > TTA	S = > L	1
	1292	431	TCC = > TTC	S = > F	1
	1481	494	CCA = > CTA	P = > L	1
*ndhD*	2	1	ACG = > ATG	T = > M	1
	878	293	TCA = > TTA	S = > L	1
*ndhF*	124	42	CTT = > TTT	L = > F	1
	290	97	TCA = > TTA	S = > L	1
	1504	502	CTT = > TTT	L = > F	1
*petB*	424	142	CGG = > TGG	R = > W	1
	617	206	CCA = > CTA	P = > L	1
*psaB*	88	30	CTT = > TTT	L = > F	1
	193	65	CTT = > TTT	L = > F	1
	422	141	TCT = > TTT	S = > F	1
	430	144	CCT = > TTT	P = > F	0.86
	431	144	CCT = > TTT	P = > F	0.86
	544	182	CTT = > TTT	L = > F	1
	1090	364	CTT = > TTT	L = > F	1
	1277	426	CCT = > CTT	P = > L	1
	1279	427	CTT = > TTT	L = > F	0.86
	1546	516	CTT = > TTT	L = > F	1
	1961	654	TCT = > TTT	S = > F	1
	1993	665	CTC = > TTC	L = > F	1
	2096	699	CCT = > CTT	P = > L	1
*psbE*	110	37	TCG = > TTG	S = > L	1
	118	40	CCG = > TCG	P = > S	1
	146	49	GCC = > GTC	A = > V	1
	148	50	CTC = > TTC	L = > F	1
**Gene**	**Nucleotide Position**	**Amino Acid Position**	**Codon Conversion**	**Amino Acid Conversion**	**Score**
*rpl2*	596	199	GCG = > GTG	A = > V	0.86
*rpl20*	308	103	TCA = > TTA	S = > L	0.86
*rpoA*	887	296	TCG = > TTG	S = > L	1
*rpoB*	473	158	TCA = > TTA	S = > L	0.86
	551	184	TCA = > TTA	S = > L	1
	566	189	TCG = > TTG	S = > L	1
	593	198	GCT = > GTT	A = > V	0.86
	1289	430	ACC = > ATC	T = > I	0.86
	2426	809	TCA = > TTA	S = > L	0.86
*rpoC2*	2287	763	CGG = > TGG	R = > W	1
	3121	1041	CGC = > TGC	R = > C	0.8
	3725	1242	TCA = > TTA	S = > L	0.86
*rps2*	248	83	TCA = > TTA	S = > L	1
*rps8*	113	38	ACT = > ATT	T = > I	1
	119	40	CCG = > CTG	P = > L	1
	257	86	ACC = > ATC	T = > I	0.86
*rps14*	80	27	TCA = > TTA	S = > L	1
	149	50	TCA = > TTA	S = > L	1

### Long repeats

Repeat sequence in the chloroplast genome of *B. prionitis* were screen using REPuter programme with default settings, the programme revealed that only three types of repeats were present in the genome viz. Palindromic, forward and reverse, the complement repeat is not detected within the plastome (Table 6). The result revealed 18 palindromic repeats, 25 forward repeats and 6 reverse repeats (Table 6). Most of the repeats size are between 20 and 29 bp (78.6%), followed by 10–19 bp (10.20%) whereas 40-49 bp are the least (4.08%). In all, there are 49 number repeats in *B*. *priniotis* plastome. In the first location, 65.30% of the repeats are contained in the non coding region; this is comparable to the cp genome of *Fagopyrum dibotrys* [44]. Eight repeats were located in the tRNA (16.32%), the other 9 repeats (18.36%) are situated in the protein coding genes in particular *rpl2*, *ndhA*, *ycf1, ndhC*, and *ycf2*. Among the protein coding genes *ycf2* contained 2 forward palindromic and repeats.

**Table 6:** Repeat sequences present in the *B. prionitis* chloroplast genome

**S/No**	**Repeat size**	**Repeat type**	**Repeat Position 1**	**Repeat Location 1**	**Repeat Position 2**	**Repeat Location 2**	**E-value**
1	41	F	97,869	*rpl2*	118,701	*ndhA*	1.35E-15
2	41	P	118,701	*ndhA*	138,043	*rps12*	1.35E-15
3	34	F	45,870	IGS	45,903	IGS	2.21E-11
4	32	F	42,498	*ycf3* Intron	42,528	*ycf3* Intron	3.53E-10
5	30	P	7813	IGS-*trnS-GCU*	44,688	*trnS-GGA*	5.65E-09
6	29	F	345	IGS	71	IGS	2.26E-08
7	27	F	45,851	IGS	45,867	IGS	3.62E-07
8	26	P	87,326	*ycf2*	87,326	*ycf2*	1.45E-06
9	26	F	87,326	*ycf2*	148,601	*ycf2*	1.45E-06
10	26	P	111,582	IGS	111,582	IGS	1.45E-06
11	26	F	122,197	IGS	122,222	IGS	1.45E-06
12	26	P	148,601	*ycf2*	148,601	*ycf2*	1.45E-06
13	24	F	45,854	IGS	45,903	IGS	2.32E-05
14	23	F	43,353	IGS	97,871	IGS	9.26E-05
15	23	F	43,353	IGS	118,703	IGS	9.26E-05
16	23	P	43,353	IGS	138,059	IGS	9.26E-05
17	23	F	59,543	IGS	59,564	IGS	9.26E-05
18	23	R	65,867	IGS	65,867	IGS	9.26E-05
19	23	F	70,995	IGS	71,017	IGS	9.26E-05
20	22	F	9299	*trnG-GCC*	36,153	*trnG-UCC*	3.70E-04
21	22	P	30,692	IGS	30,692	IGS	3.70E-04
22	22	F	90,910	IGS	90,928	IGS	3.70E-04
23	22	P	90,910	IGS	145,003	*ycf2*	3.70E-04
24	22	P	90,928	IGS	145,021	*ycf2*	3.70E-04
**S/No**	**Repeat size**	**Repeat type**	**Repeat Position 1**	**Repeat Location 1**	**Repeat Position 2**	**Repeat Location 2**	**E-value**
25	22	P	93,326	IGS	93,352	IGS	3.70E-04
26	22	F	93,326	IGS	142,579	IGS	3.70E-04
27	22	F	93,352	IGS	142,605	IGS	3.70E-04
28	22	P	142,579	IGS	142,605	IGS	3.70E-04
29	22	F	145,003	*ycf2*	145,021	*ycf2*	3.70E-04
30	21	F	82	IGS	103	IGS	1.48E-03
31	21	F	7819	*trnS-GCU*	35,296	*trnS-UGA*	1.48E-03
32	21	P	35,296	*trnS-UGA*	44,691	*trnS-GGA*	1.48E-03
33	21	F	36,373	*trnfM-CAU*	66,402	*trnP-UGG*	1.48E-03
34	21	R	119,320	IGS	119,320	IGS	1.48E-03
35	20	F	3731	IGS	111,632	IGS	5.93E-03
36	20	R	30,031	IGS	30,031	IGS	5.93E-03
37	20	R	45,796	IGS	45,796	IGS	5.93E-03
38	20	R	49,989	*ndhC*	49,989	*ndhC*	5.93E-03
39	20	P	51,944	*trnV-UAC*	102,610	*trnA-UGC*	5.93E-03
40	20	F	51,944	*trnV-UAC*	133,323	*trnA-UGC*	5.93E-03
41	20	P	56,548	IGS	56,548	IGS	5.93E-03
42	20	F	57,115	IGS	57,135	IGS	5.93E-03
43	20	P	73,944	IGS	73,967	IGS	5.93E-03
44	20	P	123,426	*ycf1*	123,426	*ycf1*	5.93E-03
45	19	P	70,448	IGS	123,029	*ycf1*	2.37E-02
46	18	F	249	IGS	272	IGS	9.48E-02
47	18	P	1946	IGS	42,695	*ycf3* Intron	9.48E-02
48	18	R	7245	IGS	7245	IGS	9.48E-02
49	18	F	7884	*trnS-GCU*	35,366	*trnS-UGA*	9.48E-02

**Fig. 3:**
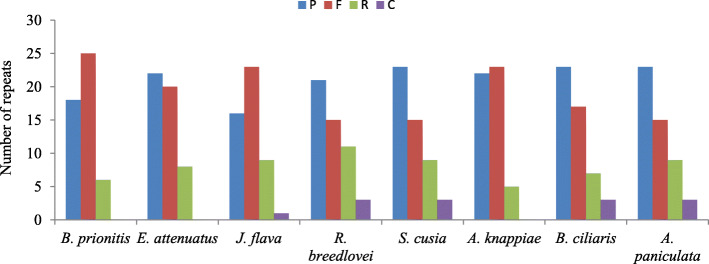
Number of different repeats in four chloroplast genome of Acanthaceae. P = palindromic, F = forward, R = reverse and C = complement

The rate of repeats among eight Acanthoideae plastomes was compared, the results indicates that complement, palindromic, reverse and forward type of repeats occurred in the plastome of *J. flava, A. paniculata*, *S. cusia*, *B. ciliaris* and *R. breedlovei*, whereas no complement repeats detected in the cp genomes of *B. prionitis*, *E. attenuatus* and *A. knappiae* (Fig. 3). *S*. *cusia, B. ciliaris* and *A. paniculata* are found to have high frequency of palindromic repeats (23) and *J. flava* is found to have the least (16). *R. breedlovei*, *S. cusia* and *A. paniculata* have15 forward repeats in their plastome and the frequency of reverse repeats is identical in the plastome of *A. paniculata, S. cusia* and *J. flava*. Complement repeat is absent in *B. prionitis, E. attenuates, A. knappiae* and is the least repeat in the plastome of *J. flava, A. paniculata, B. ciliaris, R. breedlovei* and *S. cusia*.

### Microsatellite analysis

**Fig. 4:**
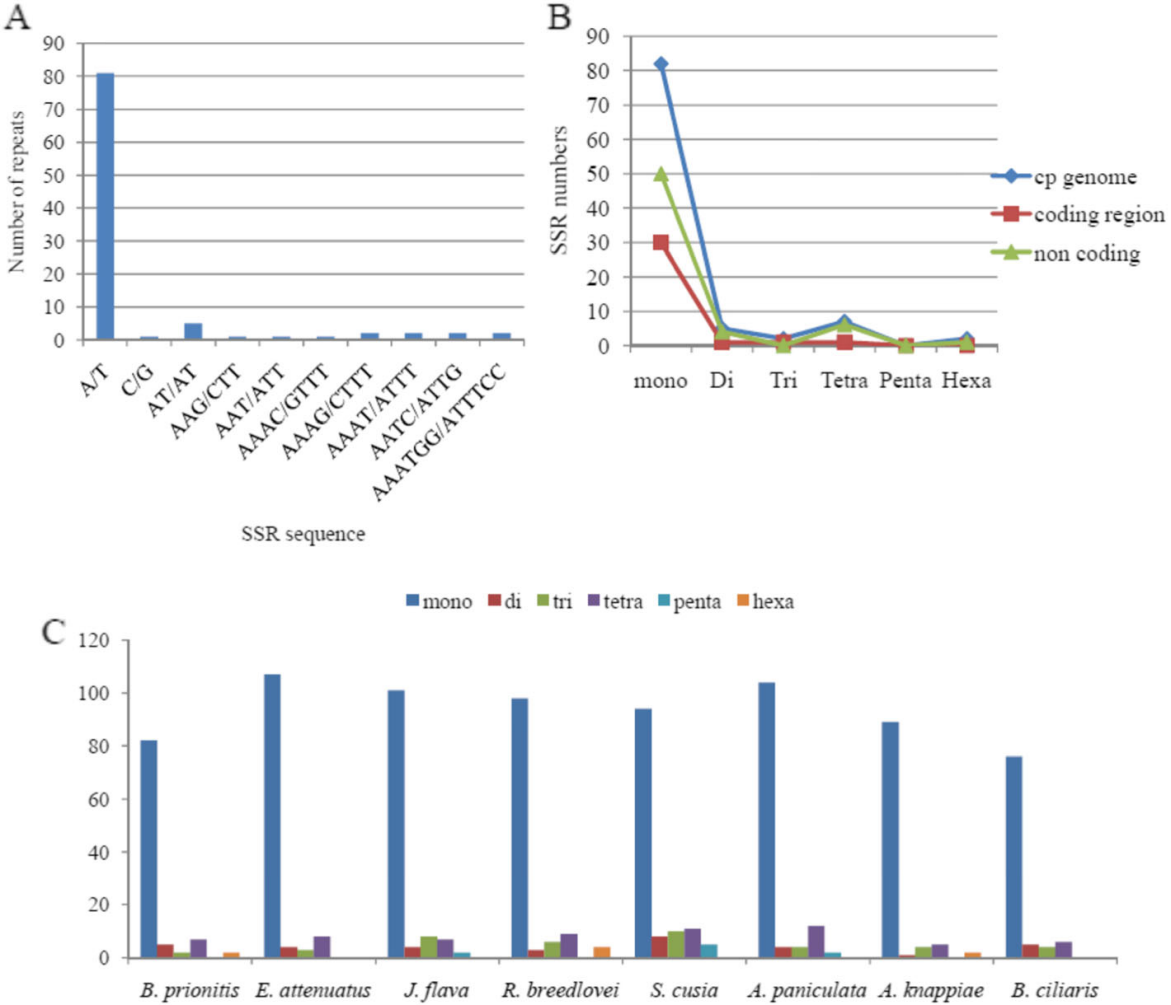
Simple sequence repeats (SSRs) types, distribution and presence in *B. prionitis* and other representatives species from Acanthoideae (**a**) Frequency of different SSR motifs in different repeat types in *B. prionitis* chloroplast genome. **b** Number of SSR types in complete genome, protein coding regions and Non coding genes. **c** Number of different SSR type in the eight chloroplast genome of Acanthoideae

Microsatellites (SSRs) are short repeat of nucleotide sequences (1-6 bp) that are distributed throughout genome. This short repeats are used as important makers for evolutionary studies of plants [45]. In this research, a total number of 98 microsatellites were identified in the chloroplast genome of *B*. *priniotis* (Table 7). Most of the microsatellites in the plastome are mononucleotide (83.67%) and majority of them are polythymine 58.53% followed by poly A (polyadenine) 40.24%, only one Poly G (polyguanine (1.21%) is present where as no poly C detected in the genome. Among dinucleotide only 5 repeats were detected, TA repeated four times and AT only once. Considering sequence complimentary, two trinucleotide AAG/CTT and AAT/ATT, four tetra AAAC/GTTT, AAAG/CTTT, AAAT/ATTT, AATC/ATTG and only one penta AAATGG/ATTTCC were detected in the genome (Fig. 4a) whereas no hexanucleotide repeat detected. The majority of the microsatellites are found in the intergenic spacer region (Fig. 4b) (62.24%) and the coding region contained the least (33.67%). The majority of the repeats were located in the large single copy region (70.40%) and the single copy region contained the lowest frequency of repeat (9.18%) in the plastome.

**Table 7:** Simple sequence repeats in the chloroplast genome of *B. prionitis*

cpSSR ID	Repeat Motif	Length (bp)	No. of Repeats	SSR start position
1	(A)8	8	22	4, 148; 11, 237; 15,924; 18,270 (*rpoC2*); 21,932 (*rpoC1*); 22,378; 44,464; 46,615; 59,100; 62,380 (*petA*); 64,357 (*psbF*); 68,879; 70,310; 70,923; 74,985; 96,279; 110,695 (*ndhF*); 113,108 (*ccsA*); 115,491 (*ndhD*); 123,247 (*ycf1*); 124,939 (*ycf1*); 126,594 (*ycf1*)
2	(A)9	9	8	3363 (*matK*); 89, 137 (*ycf2*); 28,351; 30,176; 68,148; 89,137 (*ycf2*); 112,433; 133,945
3	(A)10	10	3	6496; 70,448; 115,641
4	(G)8	8	1	58, 275 (*accD*)
5	(T)8	8	21	9447; 9599; 9655; 30,954; 35,949; 46,646; 46,783; 59,282; 59,842 (*ycf4*); 68,159; 70,217; 70,286; 73,675; 76,792 (pet D); 83,687 (rps19); 109,738 (*ndhF*); 112,390; 113,446 (*ccsA*); 114,650 (*ndhD*); 124,356 (*ycf1*); 139,668
6	(T)9	9	16	7179 (*psbK*); 12,618; 13,606; 16,227 (*rpoC2*); 35,155; 45,651; 54,687; 65,875; 67,203; 83,719 (*rps19*); 102,001; 122,650 (*ycf1*); 123,040 (*ycf1*); 124,647 (*ycf1*); 124,977 (*ycf1*); 146,809 (*ycf2*)
7	(T)10	10	3	42,062; 54,268 (*atpB*); 77,754 (*rpoA*)
8	(T)11	11	4	18,127 (*rpoC2*); 29,006; 43,047; 50,631
9	(T)12	12	2	7567; 11,941;
10	(T)13	13	1	81,706
11	(T(16)	16	1	30,034
12	(AT)5	5	1	19,500 (*rpoC2*)
13	(TA)5	5	4	45,796; 45,807; 45,823; 58,964
14	(ATA)4	4	1	54,322
15	(TTC)4	4	1	34,936 (*psbC*)
16	(AAAC)1	1	1	67,703
17	(AATA)1	1	1	114,259 (*ndhD*)
18	(AATC)1	1	1	121,984
19	(AGAA)1	1	1	9020
20	(ATAA)1	1	1	54,335
21	(GAAA)1	1	1	60,503
22	(GATT)1	1	1	5638
23	(AATGGA)1	1	1	99,217
24	(TTTCCA)1	1	1	136,718

The rate of occurrence of SSRs among the plastomes of the eight members of Acanthoideae was compared (Fig. 4c); the comparison indicate high frequency of mononucleotides across all the plastomes. *E. attenuatus* and *A. paniculata* had the highest number of mononucleotide with 107 and 104 respectively. Pentanucleotides were not found in the plastome of *B. prionitis*, *E. attenuatus*, *A. knappiae, B. ciliaris* and *R. breedlovei* while hexanucleotide were only present in *B. prionitis, R. breedlovei* and *A. knappiae.*

### Comparative analysis of Justicia flava chloroplast to other Acanthaceae genomes

**Fig. 5:**
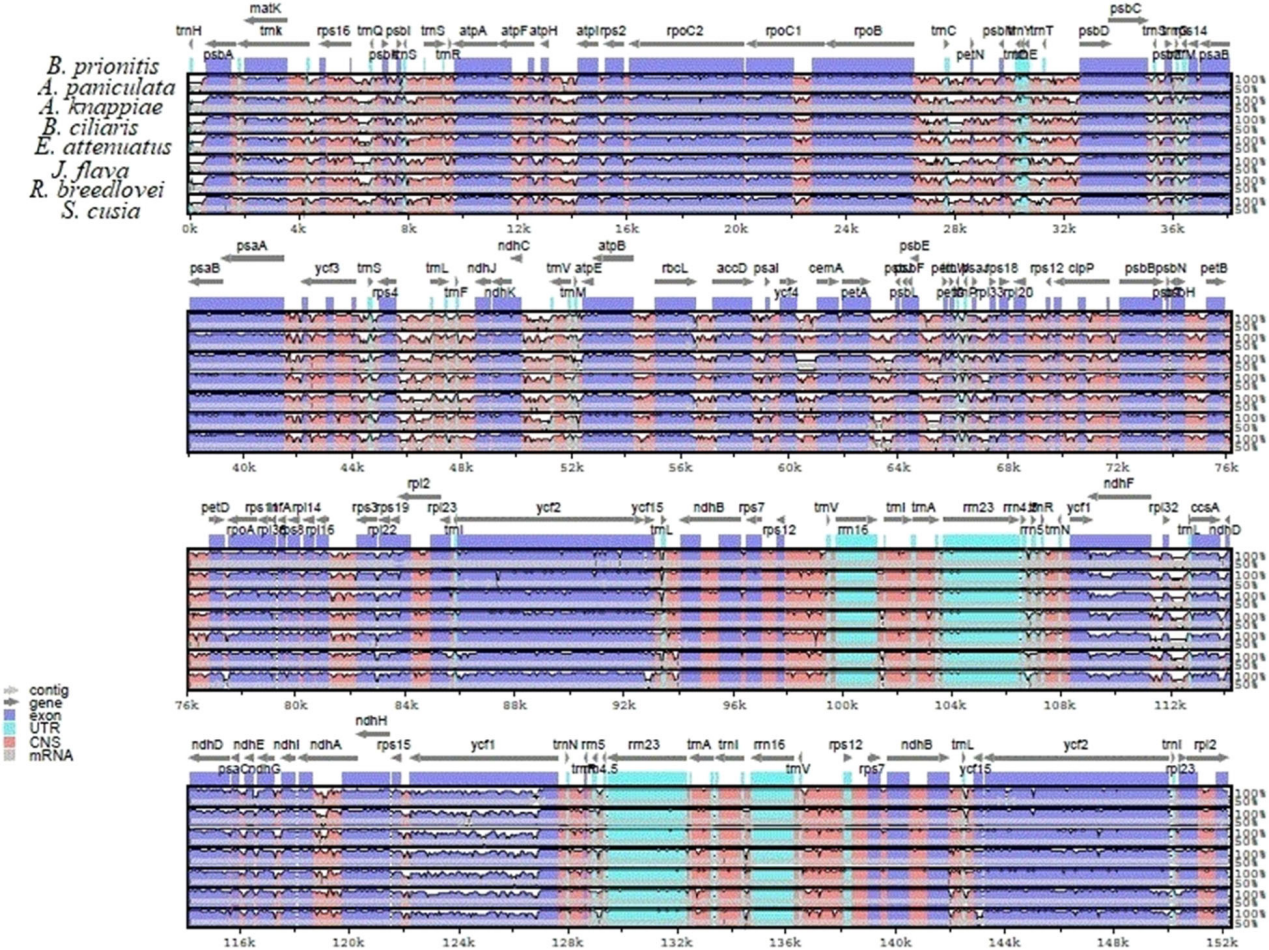
Sequence alignment of eight chloroplast genome in the Acanthaceae family performed with mVISTA using annotation of *B. prionitis* as reference. The top arrow shows transcription direction, blue colour indicatesprotein coding, pink colour shows conserved non coding sequence CNS and light green indicates tRNAs and rRNAs. The x-axis represents the coordinates in the cp genome while y- axis represents percentage identity within 50–100%

The plastome sequences of eight Acanthaceae species namely (*B. prionitis, J. flava, B. ciliaris, A. paniculata, E. attenuatus, R. breedlovei*, *A. knappiae* and *S. cusia* were compared. To check the level of nucleotide sequence variation between the sampled plastomes of Acanthoideae species, the programe mVISTA was used to aligned the sequences with the annotation of *B. prionitis* as reference. Result of the alignment indicates that the plastomes are extremely conserved, however some level of variations were detected. The pair of the iverted repeat is highly conserved than the small single copy region and large single copy region. Additionally, the protein-coding genes are highly conserved than the non coding region, mostly the integernenic spacer regions. The intergenic spacer regions with high level of variation within the gemone are *trnL* – *trnA, trnH-GUG* – *psbA*, *trnC* – *petN*, *trnL* – *trnF*, *accD* – *psaI, rps12*- *trnV*, *rps15* – *ycf1, rps16* – *trnQ* (Fig. 5). The protein coding genes that showed sequence divergence are *ycf2, psbL, atpE, rbcL, petB, petA, and atpF.*

**Fig. 6:**
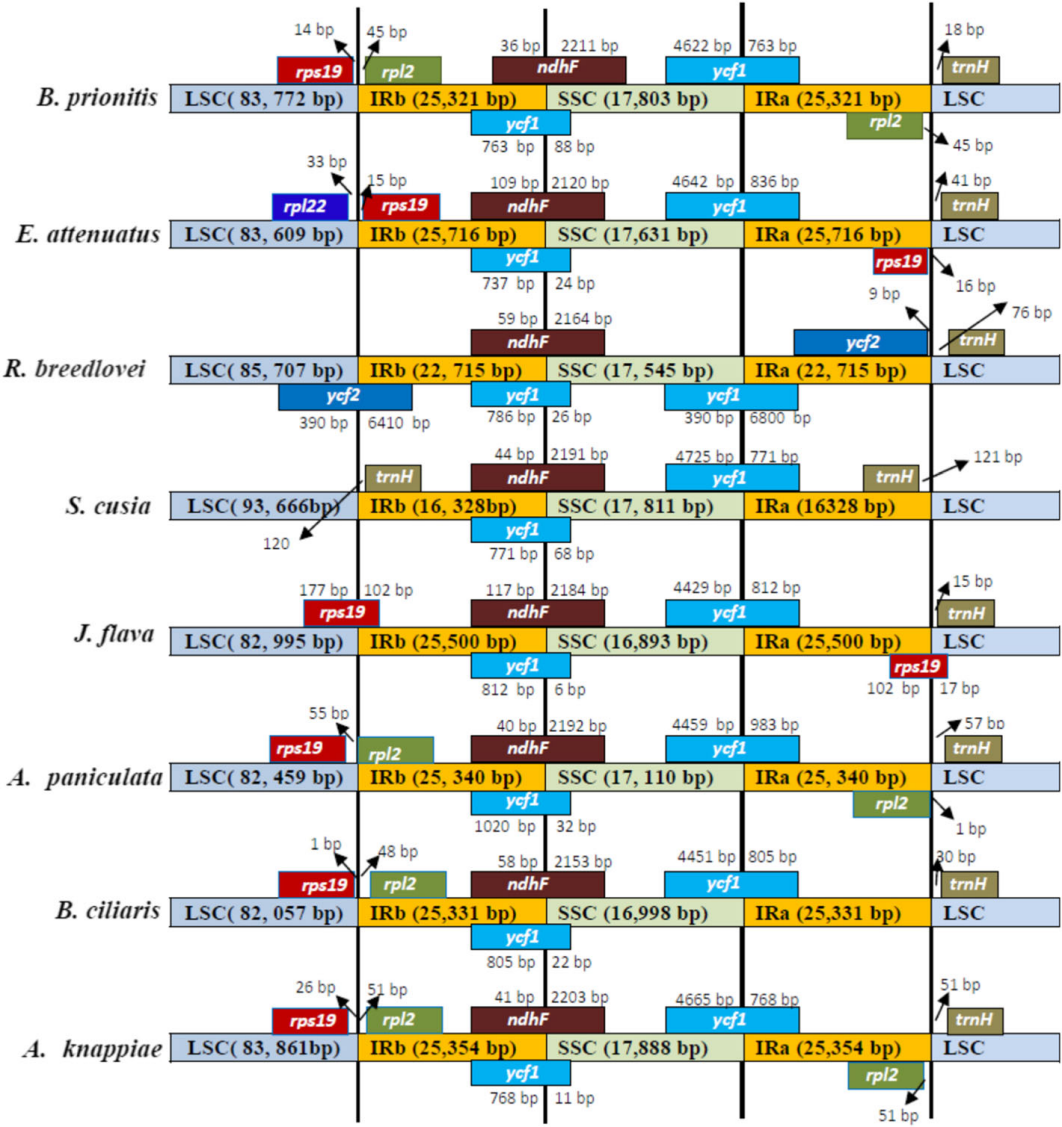
Comparison of the borders of the IR, SSC and LSC regions among eight chloroplast genome of Acanthaceae

The plastome sequence of flowering plant is reported to have generally been conserved [46], although there is a little variations in size and boundries of the single copy and inverted repeats as a results of the evolutionary happenings such as contraction and expansion in the plastome architecture [47, 48]. The comparison between the invterted repats and single copy regions boundries in the eight plastome of Acanthaceae (*B. prionitis, B. ciliaris, A. paniculata, E. attenuatus, R. breedlovei*, *J. flava, S.cusia* and *A. knappiae* are presented in (Fig. 6). There is a little variation in the boundaries of the IR-SSC and IR-LSC of the plastomes (Fig. 6),the *rps19* is located in LSC region of *B. prionitis, B. ciliaris, A. paniculata* and *A. knappiae.* The following genes *trnH, rps19, ycf1* and *ndhF* are located at the junction of IR-SSC and IR-LSC of *J. flava* and *E. attenuatus* plastomes slightly variation in number of nucleotides (Fig. 6). In the SSC/IRb border of the eight plastomes, *ycf1* and *ndhF* genes are found. Positioning of *ycf2* gene in the IRb/LCS border is observed only in the genome of *R*. *breedlovei* where as *E. attenuatus* plastome also has distinctive structural variation of having *rpl22* in junction of IRb/LSC. The gene *ndhF* was found to have 36 bp, 109 bp, 40 bp and 41 in the IRb region in *B. prionitis, E. attenuatus, A. paniculata* and *A. knappiae* respectively where as *trnH* of *E. attenuatus* and *J. flava* is positioned at IRa/LSC border .

### Divergence of protein coding gene sequence

**Fig. 7:**
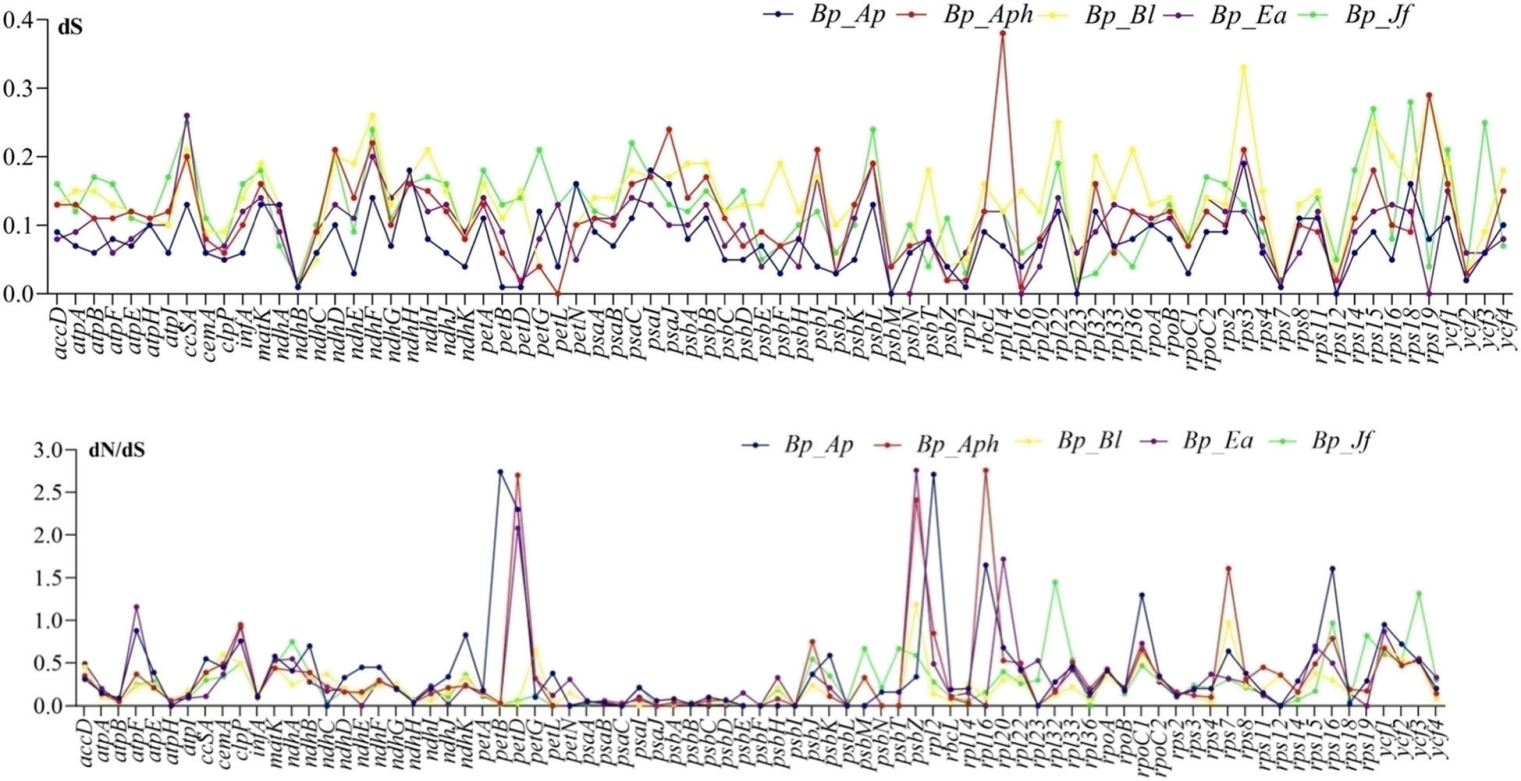
The synonymous (dS) and dN/dS ration values of 78 protein coding genes from four Acanthaceae cp genomes (*Bp*: *B. prionitis*; *Ap*: *A. paniculata*; *Aph*: *A*. *knappiae*; *Bl*: *B. ciliaris; Ea*: *E. attenuatus*; *Jf*: *J*. *flava*

The dN/dS ratio and rates of nonsynonymous (dN) substitution and synonymous (dS) were calculated using DNAsp among the plastome of eight species of Acanthoideae to detect the protein-coding genes that were under selective pressure. The results revealed that the dN/dS ratio is < 1 in most of the genes with the exception of *atpF, petD, psbZ* and *rpl20 of B. prionitis* vs *E. attenuatus*, *petB*, *petD, rpl16, rpoC* and *rps16* of *B. prionitis* vs *A. paniculata*, *petD, psbZ, rpl16, rps7*of *B. prionitis* vs *A*. *knappieae, psbZ* of *B. ciliaris*, *rpl32*and *ycf3* of *B. prionitis* vs *J. flava* having 1.16, 2.08, 2.76 and 1.72, 2.74, 2.30, 2.71, 1.65, 1.30 and 1.61, 2.70, 2.41, 2.76 and 1.61, 1.19, 1.45 and 1.32 respectively (Fig. 7). This shows that the majority of the genes undergo negative selection only few of them were under positive selection. The values of synonymous (dS) rate ranged from 0.01 to 0.38 in all the genes (Fig. 7). Some of the genes including *psaJ, atpH, ndhC, psaI, psbE, rpl2, psbH, psbI, psbL* and *psbF* showed no nonsynonymous changes.

### Phylogenetic analysis

**Fig. 8:**
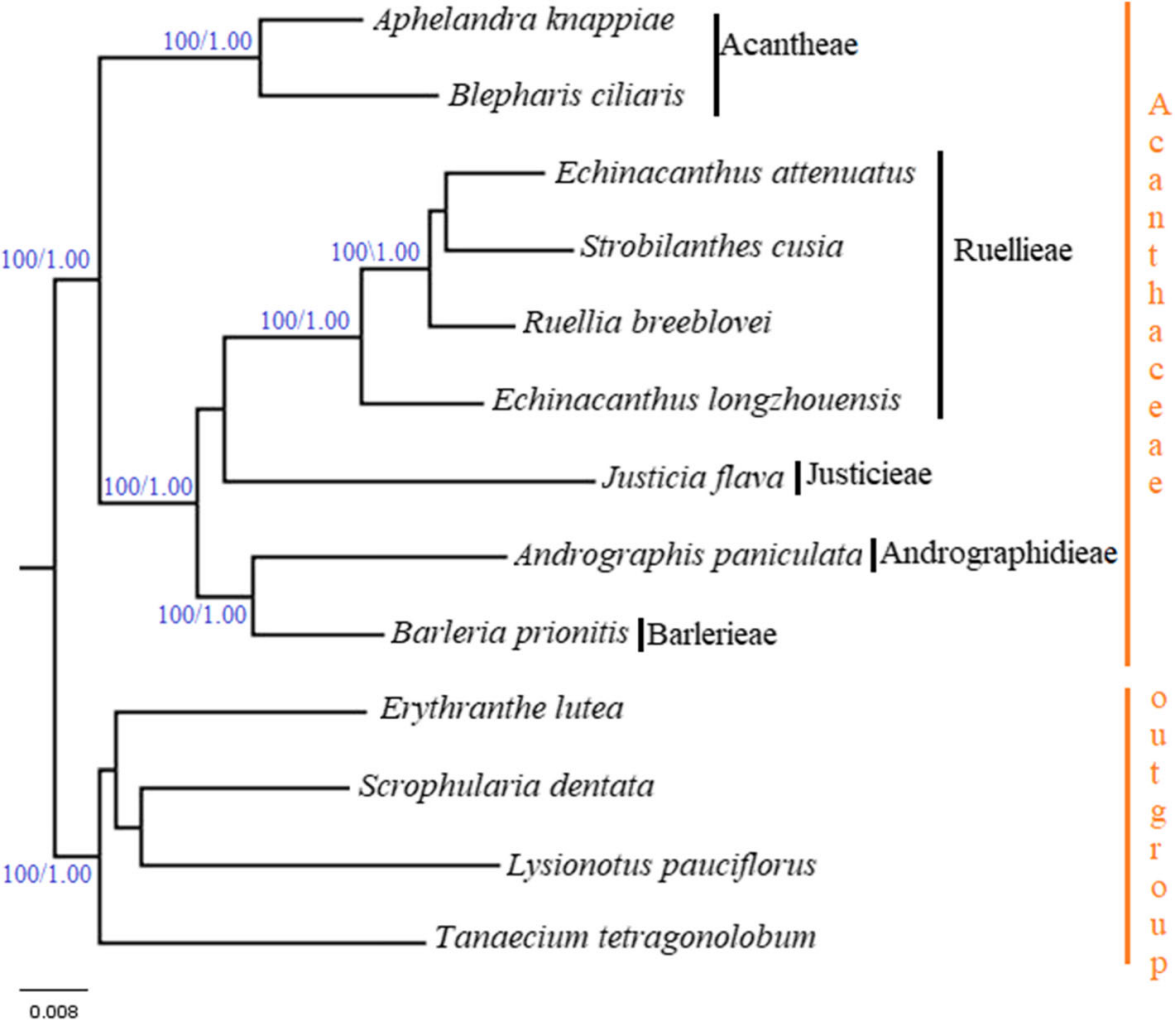
Phylogenetic tree reconstruction of 9 taxa based on the complete chloroplast genome using Bayesian Inference (BI) and Maximum Parsimony (MP) methods showing relationship within the nine species of Acanthaceae. The numbers in the branch nodes represent bootstrap percentage (BP)/posterior probability (PP)

To determine the phylogenetic relationship and tribal positions of the nine species of Acanthaceae, we used the plastome of the eight species to reconstruct phylogenetic tree. The phylogenetic analyses were performed using Maximum likelihood and Bayesian inference (BI) with *Erythranthe lutea, Scrophularia dentate, Lysionatus pauciflorus and Tanaecium tetragonolobum* as outgroup. The resulting tree from Bayesian inference (BI) and Maximum likelihood analyses were congruent with high support PP, 1.0 and MP, 100 in all relationships (Fig. 8). All the nine species clustered in one clade with strong support and are divided into two major sub clades. Sub clade 1 which is monophyletic includes *A*. *knappiae* and *B. ciliaris* (Acantheae) is sister to large clade 2 containing Ruellieae, Barlerieae, Justicieae, Andrographideae. Within the second clade Justicieae and Ruellieae are sister taxa as well as Barlerieae and Andrographideae.

## Discussion

In this study, we sequenced the plastome sequence of *B. prionitis* using Illumina sequencing technology. This is a new approach of obtaining cp genome without prior isolation of the cpDNA and it has been used in several studies. The analysis of the cp genome revealed that the genome has a quadripartite structure; with a pair of inverted repeats regions (IRa and IRb) separated by small single copy region (SSC) and large single copy region (LSC). The organization and structure of the *B. prionitis* cp genome is similar to other sequenced Acanthaceae cp genomes [49, 50]. Notably, there is high variation in terms of genome size and organization between *B.prionitis* and *S. cusia*, this is as a result of IR contraction. The size of the genome 152,217 bp is comparable to other sequenced cp genome of Acanthaceae species, longer than *A. paniculata* [51], *R. breedlovei* [50] and *S. cusia* [52] shorter than *E. attenuatus* [49]. The size of the genome in all the studied species is relevant to variation in the LSC region. The cp genome of *B. prionitis* was found to posses 38.3% GC content, as in *S.cusia* [52]. Additionally, *rps12* was recognized as trans-spliced gene, this was reported in other species [52–54]. The arrangement and gene contents of the *B. prionitis* cp genome is similar to other sequence cp genome of Acanthaceae [50, 51] but is different with that of *S. cusia* which has *trnH-GUG* in the inverted repeat regions and *ycf2* in the large single copy [52]. Some of the genes in the cp genome of *B. prionitis* start with ACG, GTG and ATC codon, this phenomenon have been reported in angiosperm chloroplast genome [36, 37, 55].

Repeat elements present in cp genome are correlated with the genome recombination and rearrangements [56, 57]. The cp genome of *B. prionits* is found have low number of repeats compared to sequenced Acanthaceae plastome [47, 51, 52]. Acanthaceae plastomes contained low repeats compared with other angiosperm cp genome. Most of the repeats were located in the non coding region and *ycf* genes (*ycf1* and *ycf2*), this has been commonly observed in plastome of angiosperms [58]. Chloroplast microsatellites (cpSSRs) are short repeat in chloroplast genome inherited from a single parent, hence are often used as molecular makers in evolutionary studies such as genetic diversity, they also play role in identification of species [59–61]. cp microsatellites analysis, reveal total number of 98 SSRs in the cp genome of *B. prionits* of which most are mononucleotides, A and T. Poly A and T are reported to be the most abundant repeat in cp genome of plants [62–64]. Most of the cpSSRs are located in the non coding region whereas few are located in the protein coding genes region. The microsatellite detected in this study will be useful in evolutionary studies of the genus *Barleria* as well as identification and conservation of the genus.

Variation in size among cp genome is as a result of contraction and expansion of the inverted repeats (IRs) [65]. Contraction and expansion in IRs region were observed in the cp genome *B. prionitis* and other sequenced Acanthaceae. The size of the inverted repeats ranges from 16, 328 bp in *S. cusia* to 25, 761 bp in *E. attenuatus*. Despite the similar lengths of the IR regions of *B. prionitis* and the other Acanthaceae species with the exception of *S. cusia* some level of expansion and contraction were observed. There are variation in the border of IR-SC region among the eight species compared, we identified six type of junctions based on the position of *rps19*, *rpl2* and *trnH*, which occur as a result of contraction and expansion in the inverted repeat region. Type I occurs in three species *B. prionitis*, *A*. *knappiae* and *A. paniculata*, one of the duplicated *rpl2* is located in the LSC region while the other is in the IRb region whereas only 1 *rps19* is present in the LSC region. Type II was found in *E. attenuatus*, here the two *rps19* are located in the inverted repeat regions (IRa and IRb) and the *rpl22* gene is located in the LSC region. Type III pattern occurs in *S. cusia* and is characterized by having *trnH-GUG* duplicated in the inverted region. Type IV has no genes in the IRb/LSC border and was only found in *R. breedlovei*. In type V which is observed in the genome *J. flava*, some part of the *rps19* gene are located in the inverted repeat region while some are located in single copy region, another remarkable observation is that the two *rps19* are of unequal length. The last pattern, type VI occurs in *B. ciliaris* and is characterized by having *rps19* in the LSC region and *rpl2* in the IRb region. All the genomes have *ndhF* in the IRb/SSC border as well as *ycf1* in the SSC/IRa border. It is observed that there is extension of inverted repeat into the single copy region in genome of *S. cusia* which made the LSC region to have length of 93, 666 bp. Despite the conserve nature of the cp genome, some variation could be detected [65]. The positioning of *ycf1* gene in IRb, is considered a pseudogene in many flowering plant plastomes. In addition, the stop codon is absent in the *ycf1* gene sequence and this result to the differences in the distribution of genes in single copy and inverted repeat borders. The result of the comparative genome analysis using mVISTA revealed that the genome is relatively conserved with some degree of variation, which mostly occurs in the non coding region as a result of insertion and deletion. The results of the alignment showed no considerable structural rearrangements, like gene relocation or inversion were detected in the plastomes. The structural rearrangement was detected in the cp genome of *S. cusia*. DNA barcodes are sequences in the genome unique to particular taxa and are used as reliable tools for identification of plants and resolving phylogenetic relationship [65, 66]. The alignment of the eight cp genome reveals variable regions which includes *trnH-GUG* – *psbA*, *rps16* – *trnQ*, *trnC* – *petN*, *accD* – *psaI*, *clpP* intron, *trnL* – *trnF*, *rps15* – *ycf1*, *rps12*- *trnV*, *trnL* – *trnA*, *atpE, atpF, rbcL*. These regions will be used as makers for identification of the sampled Acanthaceae species as well as resolving phylogetic relationships in the family. Most of the variable regions are located in the single copy region particularly the large single copy, this is consistent in most angiosperms.

Synonymous (dS) and non synonymous (dN) substitution rate as well as dN/dS ration were calculated to evaluate sequence divergence and purifying selection in the protein coding genes. The result indicates low sequence divergence in most of the genes (dS < 0.1). The dN/dS analyses show that most of the protein coding genes were under negative selection, only few genes *(atpF, petD, psbZZ, rpl20, petB, rpl16*, *rps16, rpoC, rps7, rpl32* and *ycf3*) were under positive selection (dN/dS > 1), comparable findings were reported for other plastomes [66–68].

Complete chloroplast genome is a good resource for inferring evolutionary and phylogenetic relationships [69–71]. Many researchers have used the plastome sequence to resolve phylogenetic relationships at various taxonomic levels [72, 73]. Until this study, the phylogenetic relationships and tribal classification of Acanthaceae was evaluated using only few genes and the tribal classification is still required to be clarified. In this study, we used the cp genome of nine species representing the four major tribes of the Acanthoideae and reconstructed phylogenetic relationships based on maximum parsimony and Bayesian inference methods. The resulting phylogenetic tree from the two methods showed the same topology with high resolution values at the clades. The result of this study based on nine Acanthaceae taxa confirm that Acanthoideae (the retinaculate clade) are monophyletic and also confirm the sister relationship between Acantheae (non cystolith clade) and the cystolith clade, this has been reported earlier [11–13, 19] . The phylogenetic tree showed Justicieae and Ruelliae are sister taxa as reported previously [19] therefore should be regarded as separate tribes not as Justicieae or Ruelliae because the species within these two taxa are paraphyletic. The sister relationship between Andrographideae and Barlerieae is also confirm. Andrographideae and Barlerieae were placed in the tribe Justiceae as sub tribes [35, 74]. Recently Scotland and Vollesen classified all species with cystolith under the tribe Ruellieae placing *Andrographis*, *Barleria* and *Justicia* under the sub tribes Andrographinae, Barleriinae and Justiciinae respectively. Our findings suggested that Andrographideae, Justicieae and Barlerieae should be treated as tribes not sub tribes.

## Conclusion

In this study, we sequenced and reported the complete chloroplast genome of *B. prionitis*, providing valuable plastome genomic resources for the species. The plastome of *B. prionitis* has a typical gymonosperm cp genome structure and is comparable to other cp genome of Acanthaceae. Simple sequence repeats that will be used for evolutionary studies within Barleria were identified. The genome comparative analyses of 9 Acanthaceae reveal variable hotspot that could be used to develop DNA barcode for the identification of the species. These hotspots will also be useful in phylogenetic relationship studies of the family Acanthaceae. The study also reveals that only few genes were under positive selection. The findings of the confirmed the tribal position of major genera within Acanthoideae and suggested that Andrographideae, Justicieae and Barlerieae should be treated as tribes not sub tribes.

## Methods

### Plant material and DNA extraction

Plant material was collected from Makkah Taif road, Saudi Arabia (39^0^ 20′ 0.30″E, 21^0^ 45′ 33.68″N) and identified by the curator of King Abdulaziz University Herbarium, Dr. Dhafer A. Alzahrani, the voucher specimen was deposited in the herbarium of King Abdulaziz University, Jeddah, Saudi Arabia, with voucher specimen number KAU22534. Total genomic DNA was extracted from leaves using Qiagen DNA extraction Kit according to manufacturer’s protocol.

### Library construction, sequencing and assembly

The genomic DNA was sequenced using Illumina Hiseq 2500 platform (Novogene Technologies, Inc. Beijing, China). Raw data reads were filtered by PRINSEQ lite Ver0.20.4 [75] to get clean reads (5GB). The cp genome was assembled from the high quality clean reads using NOVOplasty2.7.2 [76] with kmer 39 using the cp genome of *Ruellia breedlovei* (KP300014.1) as reference and *ndhF* from *B. prionitis* (U12653) as seed.

### Gene annotation

Dual Organellar GenoMe Annotator (DOGMA) [77] was used to annotate the genes in plastome followed by manual adjustment of the positions of start and stop codons. TrNAscan-SE2.0 [78] was used to verify tRNA genes. Organellar Genome Draw (ORGDRAW) [79] was used to circular map of plastome. The complete chloroplast genome sequence of *B. prionitis* was submitted to GenBank (Accession number MK548575).

### Sequence analysis

Relative synonymous codon usage values (RSCU), base composition and codon usage were analyzed using MEGA 6.0. PREP suite [80] with cutoff value of 8.0 was used to predict the RNA editing sites in the plastome.

### Repeat analysis in *B. prionitis* chloroplast genome

MIcroSAtellite (MISA) [81] was used to identify the simple sequence repeats (SSRs) with the following parameters: eight for mononucleotides, five for dinucleotides, four trinucleotides and three for tetra, penta, hexanucleotides SSR motifs. Long repeats analysis was done using the program REPuter (https://bibiserv.cebitec.uni-bielefeld.de/reputer) [80] with default parameters.

### Genome comparison

mVISTA [82] was used to compare the plastome using the annotation of *B. prionitis* as reference in the Shuffle-LAGAN mode [83].

### Characterization of substitution rate

To detect the genes that were under selective pressure, DNAsp v5.10.01 [84] was used to analyze the synonymous (dS), nonsynonymous (dN) and dN/dS value of all the protein coding genes in sampled Acanthoideae species.

### Phylogenetic analysis

For phylogenomic analysis, the cp genomes of Acanthoideae species deposited in the GenBank were recovered (Table 8). The plastome of four species of the order lamiales were also downloaded and set as out groups (Table 8). The downloaded sequences and cp genome of *B. prionitis* were aligned with MAFFT v.7 [85] and analyzed using Maximum parsimony with (PAUP version 4.0b10) [86] and Bayesian Inference with MrBayes version 3.2.6 [87].. To select the suitable model for Bayesian analysis jModelTest 3.7 [88] was used.

**Table 8:** Accession numbers of plastomes analysed in the study

Name of species	Accession Number
*Aphelandra knappiae*	NC_041424.1
*Blepharis ciliaris*	NC_046601.1
*Echinacanthus attenuatus*	NC_039762.1
*Strobilanthes cusia*	NC_037485.1
*Ruellia breedlovei*	KP300014.1
*Justicia flava*	NC_044862.1
*Andrograhis paniculata*	NC_022451.2
*Barleria prionitis*	MK548575.1
*Erythranthe lutea*	NC_030212.1
*Scrophularia dentata*	NC_036942.1
*Lysionotus pauciflorus*	NC_034660.1
*Tanaecium tetragonolobum*	NC_027955.1

## Data Availability

All data generated or analysed during this study are included in this published article and the complete chloroplast genome sequence of *Barleria prionitis* is deposited in the genbank with I. D no: MK548575. The accession numbers corresponding to the additional datasets used and analysed in this study can be found in Table 8. These were retrieved from National Center for Biotechnology Information database.

## Abbreviations

BI: Bayesian Inference
CNS: Conserved non coding sequence
Cp: Chloroplast
dN: Non synonymous
DNA: Deoxyribonucleic acid
dS: Synonymous
IGS: Intergenic spacer
IR: Inverted repeat
LSC: Large single copy region
MP: Maximum Parsimony
BP: Bootstrap percentage
NGS: Next generation sequencing
PCR: Polymerase chain reaction
PP: Posterior probability
RSCU: Relative synonymous codon usage
SSC: Small single copy region
SSR: Simple sequence repeats
